# **Characterization and genomics of phage Henu2_3 against K1** ***Klebsiella pneumoniae*** **and its efficacy in animal models**

**DOI:** 10.1186/s13568-025-01919-0

**Published:** 2025-07-30

**Authors:** Qiming Li, Jiaqi Li, Yanyang Zhao, Shuai Guo, Mengzhe Liu, Xiaoyu Shi, Li Wang, Zhigang Liu, Tieshan Teng

**Affiliations:** 1 Henan Province EngineeringTechnology Research Center of Rapid-Accuracy Medical Diagnostics, Department of Clinical Laboratory, The First Affiliated Hospital of Henan University, Henan University, Kaifeng, 475000 China; 2 The Jointed National Laboratory of Antibody Drug Engineering, Henan University, Kaifeng, 475000 China; 3 Department of Microbiology, College of Basic Medical Sciences, Henan University, Kaifeng, 475000 China

**Keywords:** *Klebsiella* phage, Phage therapy, Bacteriophage, K1-type *Klebsiella pneumoniae*

## Abstract

The emergence and spread of multidrug-resistant and super-resistant bacterial strains have positioned phage therapy as a highly promising approach for future infection treatments. *Klebsiella pneumoniae*, a leading cause of clinical infections and hospital-acquired diseases, is responsible for over 95% of infections within the *Klebsiella* genus. Phages targeting *K. pneumoniae* exhibit remarkable diversity, with numerous isolates identified against various serotypes of this pathogen. In this study, we described the isolation and characterization of a novel bacteriophage from hospital sewage, Henu2_3, which targeted clinical isolates of K1-type *K. pneumoniae*. Transmission electron microscopy revealed that phage Henu2_3 possessed an icosahedral head and podovirus morphotype. The phage genome comprises 42,878 base pairs with a G + C content of 53.97%, encoding 54 putative open reading frames. One-step growth curve analysis demonstrated that phage Henu2_3 has a latency period of 10 min and an average burst size of 215 phage particles per infected cell. Additionally, Henu2_3 exhibited remarkable stability, tolerating temperatures up to 60℃ and maintaining maximum viability across a broad pH range of 4 to 12. In vivo infection models demonstrated that phage Henu2_3 markedly enhanced animal survival and decreased bacterial burden in target organs. These properties highlight its potential as a therapeutic agent against *K. pneumoniae* infections.

**Supplementary Information:**

The online version contains supplementary material available at 10.1186/s13568-025-01919-0.

## Introduction

Infections caused by drug-resistant bacteria represent one of the most formidable challenges in clinical treatment. The ESKAPE pathogens - *Enterococcus faecium*, *Staphylococcus aureus*, *Klebsiella pneumoniae*, *Acinetobacter baumannii*, *Pseudomonas aeruginosa* and *Enterobacter* spp. - were initially identified as critical multidrug-resistant bacteria for which effective therapies were rapidly needed (Miller and Arias 2024). Among these, clinical infections caused by multidrug-resistant (MDR) gram-negative bacteria have led to an increasing number of fatalities, exacerbating the already daunting challenges in treatment (Sakalauskiene et al. 2025). *K. pneumoniae* stands out as one of the most prevalent gram-negative pathogens in clinical settings, capable of causing suppurative inflammations such as pneumonia, metritis, and mastitis (Wyres et al. 2020). Based on their virulence characteristics, they are currently classified into two types: classical *K. pneumoniae* (cKP) and hypervirulent *K. pneumoniae* (hvKP). Notably, hvKP exhibits significantly higher propensity for establishing polymicrobial infections at distant anatomical sites (e.g., liver abscesses, endophthalmitis) through hematogenous spread, a feature rarely observed in cKP (Russo and Marr 2019). In the arsenal against bacterial infections, colistin is regarded as the “last line of defense” against drug-resistant gram-negative bacteria (Khondker and Rheinstadter 2020). The antimicrobial mechanism of colistin typically involves binding to lipid A of the lipopolysaccharide (LPS) in the outer membrane of gram-negative bacteria, disrupting the integrity of the bacterial cell membrane and leading to bacterial death (Andrade et al. 2020). However, in recent years, due to the misuse of antibiotics, colistin-resistant gram-negative strains have emerged. Among the current resistant strains, the plasmid-borne colistin resistance gene mcr-1, which encodes phosphoethanolamine transferase, has spread globally, threatening the efficacy of colistin (Xiaomin et al. 2020). Bacterial resistance has become a critical issue in global public health, with statistics showing that approximately 7 million people worldwide die from bacterial infections each year, with resistant bacterial infections being a major cause of death. Given the slow progress and reduced investment in the development of new antibiotics, it is imperative to seek and develop alternative therapeutic strategies to address bacterial resistance.

Bacteriophages are a broad category of viruses capable of infecting microorganisms such as bacteria, actinomycetes and spirochetes (Faith et al. 2024; Hatfull 2020). Having co-evolved with bacteria over hundreds of millions of years, they are natural predators of bacteria. As viruses, phages often undergo mutations during their rapid replication process, leading to an astronomical diversity of phages in nature (Chevallereau et al. 2022). Phages can be found in a variety of ecological environments, with an estimated population of up to 10^31^, approximately ten times the number of bacteria (Hendrix et al. 1999). As early as 1919, shortly after the discovery of phages, when knowledge of their biological characteristics was still limited, phages were employed to treat bacterial infections (Sahu et al. 2023). The discovery and rapid widespread application of antibiotics led to a stagnation in the use of bacteriophages for pathogen control. However, as bacterial resistance has become increasingly severe, phage-based alternative therapies have regained significant attention. Phage therapy, a non-antibiotic technique for treating bacterial infections, has regained popularity in recent years. Compared to antibiotics, phage infections are specific and do not affect the normal flora in human hosts (Liang et al. 2023). Moreover, the use of a phage cocktail, a mixture of several different phages, can minimize the likelihood of bacteria developing phage resistance (Gou et al. 2025).

Phages are currently widely utilized across various fields, including biological genetic engineering, the medical sector, livestock farming, agricultural cultivation, and aquaculture (Dhowlaghar and Denes 2024). Since the direct in vivo application of phages, successful treatments have been achieved for infections caused by drug-resistant pathogens such as *Mycobacterium abscessus*, *K. pneumoniae*, and *P. aeruginosa* (Dedrick et al. 2019; Little et al. 2022; Nick et al. 2022; Wu and Zhu 2021). The specificity between phages and their host bacteria makes the selection of appropriate phages essential for the effective treatment of bacterial infections. The capsular antigen of *K. pneumoniae* is one of its key virulence factors, primarily composed of capsular polysaccharides (CPS). These polysaccharides form a mucoid layer on the bacterial surface, protecting the bacteria from the host immune system attacks, such as phagocytosis by immune cells and the killing effects of the complement system. The capsular antigen, also known as the K antigen, exhibits significant diversity, which serves as the basis for serotype classification of *K. pneumoniae* (Choi et al. 2020). According to traditional serological classification based on the types and structural variations of the capsule, *K. pneumoniae* is divided into at least 79 distinct serotypes (Pan et al. 2015). CPS serve as the primary receptor for most *Klebsiella* phages, and variations in their structure significantly influence phage adsorption and infection (Cai et al. 2019). The specific chemical composition and configuration of the CPS determine the ability of phages to recognize and bind to the bacterial surface (Dunstan et al. 2023). Changes in the capsular structure, such as modifications in sugar composition or glycosidic linkages, can either enhance or hinder phage attachment, thereby affecting the efficiency of phage infection (Cai et al. 2019). This interaction highlights the critical role of CPS in the initial stages of phage-host recognition and the subsequent infection process. Therefore, establishing a comprehensive library of *Klebsiella* phages capable of infecting different serotypes is of paramount importance for the treatment of *K. pneumoniae* infections.

To obtain phages capable of specifically infecting hvKp, we used K1-type *K. pneumoniae* as the host strain for phage isolation and identification. In this study, we isolated a bacteriophage from sewage samples collected at the drainage system of The First Affiliated Hospital of Henan University. The phage Henu2_3 formed clear, halo-free plaques on double-layer agar plates. Through evaluation of the lytic stability and genomic characteristics of bacteriophage Henu2_3, along with its antibacterial efficacy both in vitro and in vivo, we demonstrated that Henu2_3 represented a promising candidate for treating *K. pneumoniae* infections.

## Materials and methods

### Bacterial strains and cultures

*K. pneumoniae*, *E. coli*,* A. baumannii* and *P. aeruginosa* isolates were grown in Luria Bertani broth (LB) in shaking incubator set at 220 r.p.m. and 37℃. All the isolates were identified through 16S rRNA sequencing using universal primers 27F (5’-AGAGTTTGATCCTGGCTCAG-3’) and 1492R (5’-TACGGCTACCTTGTTACGACTT-3’). The 16 S rRNA sequence of *K. pneumoniae* Kp1049 has been included in the supplementary materials. The K-type of *K. pneumoniae* were identified by *wzi* gene using the primers of wzi_for2 (5’-GTGCCGCGAGCGCTTTCTATCTTGGTATTCC-3’) and wzi_rev (5’-GAGAGCCACTGGTTCCAGAATTACCGC-3’) (Brisse et al. 2013). The strain *K. pneumoniae* Kp1049 was deposited in the China Center for Type Culture Collection (CCTCC) with the accession number CCTCC PB 2,025,034. This phage Henu2_3 can be obtained by contacting the China Center for Type Culture Collection or the corresponding author.

### Isolation and purification of phage Henu2_3

The phage Henu2_3 was isolated and purified as previously described (Zhou et al. 2025). Briefly, untreated sewage was centrifuged and filtered through a 0.22 μm filter, and the resulting supernatant was applied to a lawn of *K. pneumoniae* Kp1049. Following a 24 h incubation period, a single phage plaque was carefully extracted and transferred into phage buffer. This suspension was subsequently spotted onto a lawn of *K. pneumoniae* Kp1049. The purification process, involving the sequential plating and isolation of phage plaques, was repeated five times to ensure purity. Through this rigorous procedure, a novel lytic bacteriophage specific to *K. pneumoniae* was successfully isolated and purified, designated as *Klebsiella* phage Henu2_3. For the amplification of phage Henu2_3, *K. pneumoniae* Kp1049 was cultured to the exponential growth phase. The phages were then introduced into LB medium supplemented with 10 mM MgSO₄ and 10 mM CaCl₂, and the culture was incubated at 37℃ with shaking at 200 rpm for 12 h. After incubation, the supernatant was collected and filtered through a 0.22 μm filter to remove bacterial debris. The phage particles were concentrated using a 100 kDa ultrafiltration tube, and the phage titer was quantified using the double-layer agar method. This approach ensured the efficient amplification and precise quantification of phage Henu2_3 for subsequent studies.

### Transmission electron microscopy

The morphology of phage Henu2_3 was characterized using transmission electron microscopy (TEM) following previously established protocols (Eckstein et al. 2021). Briefly, purified phage Henu2_3 with a high titer (10^10^ PFU/mL) was applied to copper grids (Gilder Grids). The samples were then negatively stained with 2% uranyl acetate to enhance contrast. Imaging was performed by ZHONG KE BAI CE (http://www.zkbaice.cn/, Beijing) using a JEOL JEM 1011 transmission electron microscope operating at 80 kV (JEOL, Japan). The resulting images provided detailed visualization of the phage’s structural characteristics. The measurement of the phage head diameter was achieved using the ruler tool in Adobe Photoshop CS5.

### Host range of phage Henu2_3

Fifteen strains of *K. pneumoniae* and other gram-negative bacteria were collected from different hospital ward environments and were cultured to the exponential phase. Bacteria were mixed with 0.7% soft agar and overlaid onto LB solid medium to prepare double-layer agar plates. Phage suspension was then spotted onto the double-layer agar plates, followed by incubation at 37℃ for 12 h to observe plaque formation.

### Optimal MOI value monitorings

*K. pneumoniae* Kp1049 were cultured to the logarithmic growth stage, transferred to fresh culture medium at an initial concentration of 1 × 10^8^ CFU/mL and incubated with phage Henu2_3 for 5 min at different multiplicity of infection (MOI = 10, 1, 0.1, 0.01, 0.001 and 0.0001). The supernatant was removed by centrifugation at 8000 × g, and the bacterial pellets were resuspended in 5 mL of fresh LB medium, incubated on a shaker at 37℃ for 2 h, and then filtered after centrifugation at 12,000 × g. Finally, the phage titer was determined via a plaque assay. The optimal MOI for phage Henu2_3 is the infection ratio that produces the highest titer of progeny phages.

### One-step growth curve

The one-step growth curve was conducted following a previously established protocol with some modifications (Han et al. 2023). Briefly, 1 mL of phage Henu2_3 was mixed with 9 mL of exponentially growing *K. pneumoniae* Kp1049 at a MOI of 0.001. The mixture was incubated at 37℃ with shaking for 20 min to allow phage adsorption. Subsequently, the culture was centrifuged at 12,000 × g for 1 min, and the pellet was washed twice with LB medium to remove unadsorbed phages. The pellet was then resuspended in 10 mL of fresh LB medium and incubated at 37℃ with continuous shaking. Phage titers were measured at 10 min intervals over a 100 min period. The one-step growth curve of phage Henu2_3 was plotted with infection time as the horizontal axis and phage titer as the vertical axis.

### Stability analysis of phage Henu2_3

The stability of phage Henu2_3 was evaluated under different temperature conditions (4℃, 25℃, 37℃, 45℃, 60℃ and 65℃). Phage Henu2_3, with an initial titer of approximately 10^10^ PFU/mL, was incubated at each temperature for 1 h. Following incubation, the remaining phage titer was determined using the double-layer agar plate method on a lawn of *K. pneumoniae* Kp1049. To assess pH sensitivity, 100 µL of phage Henu2_3 in phage buffer was mixed with 900 µL of saline solutions adjusted to varying pH levels ranging from 3 to 13. The mixtures were incubated at 4℃ for 1 h to evaluate the effect of pH on phage stability. After incubation, the phage titer was quantified using the double-layer agar plate method on a lawn of *K. pneumoniae* Kp1049. Purified phage particles were stored at 4 °C for 8 months, with phage titers measured every 2 months to evaluate long-term storage stability. To evaluate the serum stability of phage Henu2_3, 100 µL of phage suspension was mixed with 900 µL human serum and incubated at 37℃ for 48 h, after which residual phage titers were determined by plaque assay.

### Genomic DNA extraction, sequencing and ***De Novo*** assembly of phage Henu2_3

The DNA of bacteriophage Henu2_3 was extracted using the phenol/chloroform extraction method following previously reported methods (Salih et al. 2022). Briefly, the phage stock was treated with PEG8000 and incubated at 4℃ for 24 h to precipitate phage particles. After centrifugation (10,000 × g, 10 min, 4℃), the pellet was resuspended in 1 mL of PBS. The sample was then treated with 0.5 µL DNase I (10 mg/mL) and 0.1 µL RNaseA (10 mg/mL) for residual nucleic acid degradation (37℃, 1 h). Next, 40 µL EDTA (0.5 M, pH 8.0), 50 µL SDS (10% w/v), and 2 µL proteinase K (25 mg/mL) were added. After three gentle inversions, the mixture was incubated at 56℃ for 1 h. Nucleic acids were extracted with equal volumes of equilibrated phenol and chloroform, collecting the aqueous phase. For precipitation, 1/10 volume of 3 M sodium acetate (pH 5.2) and two volumes of absolute ethanol were added, followed by incubation at -20℃ for 1 h. After centrifugation (12,000 × g, 20 min, 4℃), the DNA pellet was washed sequentially with absolute ethanol and 70% ethanol, then resuspended in sterile water. The extracted DNA of bacteriophage Henu2_3 was subjected to a whole-genome shotgun sequencing strategy, in which libraries with different insert sizes were constructed. The libraries were then sequenced using paired-end sequencing on the Illumina NovaSeq platform (second-generation sequencing technology). Raw data were quality-controlled using fastp. Subsequently, A5-MiSeq and SPAdes were employed for de novo assembly of adapter-trimmed reads to generate contigs (Bankevich et al. 2012; Coil et al. 2015). Sequences with high sequencing depth were extracted and aligned against the NCBI NT database using BLASTn to identify viral genomic sequences from the assembled contigs. The assembly results obtained from the aforementioned software were analyzed for collinearity using MUMmer to determine the positional relationships among contigs and to fill gaps between them (Kurtz et al. 2004). Subsequently, Pilon was employed to polish the genome sequence, yielding the final complete viral genome (GenBank accession number: PQ394119.1) (Walker et al. 2014).

### Bioinformatics analysis of phage Henu2_3 genome sequence

Open-reading frames (ORFs) of phage Henu2_3 were predicted using Softberry (http://www.softberry.com/). The function of ORFs were performed by BLASTp, the virulence factor, antimicrobial resistance and tRNA were analyzed by PhageScope (Wang et al. 2024). Multiple and pairwise sequence comparisons were performed using Easyfig and BLASTN, respectively (Chen et al. 2015; Sullivan et al. 2011). Gene organization diagrams were drawn in CGview (Grant et al. 2023). Intergenomic similarities were analyzed using the VIRIDIC tool with all highly similar phage genomes (Moraru et al. 2020).

### Phylogenetic analysis

Multiple sequence alignment of DNA polymerase and major capsid protein amino acid sequences from representative phages was performed using ClustalW (Li 2003). Phylogenetic reconstruction was conducted using the neighbor-joining method in MEGA7, with evolutionary distances calculated through 1000 bootstrap replicates (Kumar et al. 2016). Resulting phylogenetic trees were visualized and annotated using the Interactive Tree of Life (iTOL) v6 platform (Letunic and Bork 2024).

### Antibacterial and antibiofilm effects of phage Henu2_3 in vitro

One milliliter of *K. pneumoniae* Kp1049 in the exponential growth phase was inoculated into 100 mL of LB medium. When the optical density at 600 nm of the culture reached approximately 0.2, phage Henu2_3 was introduced at MOI values of 100, 10, 1, 0.1, and 0.01. An uninfected culture was included as a positive control. Samples were collected at 0.5 h intervals, and the OD_600_ was measured using an UV-Vis spectrophotometer to monitor bacterial growth dynamics in response to phage infection. To evaluate the destruction of biofilms by phage Henu2_3, bacterial cultures with an OD_600_ value of 0.3–0.4 were diluted 100-fold in fresh LB medium. Subsequently, 200 µL of the diluted mixture was transferred into a new 96-well plate and incubated statically at 37 °C for 20–24 h to facilitate biofilm formation. After incubation, the LB broth was carefully removed from the wells, and the biofilms were gently washed once with 1 × PBS to remove non-adherent cells. Next, 200 µL of phage Henu2_3 suspension was added to the wells and coincubated with the biofilms at 37℃ for an additional 20–24 h. The MOI for phage infection was determined based on the CFU of bacteria in the biofilm. Following coincubation, the supernatants were discarded, and the biofilms were washed twice with 1 × PBS, ensuring that the biofilm structures at the bottom of the wells remained intact. The quantification of biofilms was performed using the same methodology as described previously (Guo et al. 2024). Briefly, the remaining biofilms in the wells were stained with 100 µL of 0.1% crystal violet ammonium oxalate solution (Solarbio, G1063) for 20 min. Excess dye was carefully removed by washing 3 times with PBS until the wash solution appeared clear. To solubilize the stained biofilms, 100 µL of 33.3% (v/v) glacial acetic acid was added to each well and incubated until complete dissolution of the dye-bound pellets. The absorbance at 570 nm was then measured using a Thermo 96-well plate reader.

### Antibacterial effect of phage Henu2_3 in ***G. mellonella*** larvae infection model

*Galleria mellonella* larvae were employed as an in vivo model to assess the antibacterial efficacy of phage Henu2_3. Healthy larvae weighing approximately 0.3–0.5 g were selected, and 5 µL of *K. pneumoniae* Kp1049 at varying concentrations (1 × 10^6^ − 1 × 10^11^CFU/mL) was injected into the last proleg to determine the optimal infection dose. Each experimental group consisted of ten larvae, with three parallel replicates performed. *K. pneumoniae* Kp1049 in the logarithmic growth phase was washed with saline, and a lethal dose of the bacteria (5 µL, 1 × 10^9^ CFU/mL) was injected into *G. mellonella* larvae. One hour post-infection, 5 µL of the phage Henu2_3 suspension was administered at MOI values of 100, 10, 1, 0.1, and 0.01. The larvae were then incubated at 37 °C in the dark. A negative control group was established by infecting larvae with *K. pneumoniae* Kp1049 and treating them with PBS. The survival rates of the larvae were monitored every 24 h over a 7-day period.

For bacterial load quantification, 5 µL of a 1 × 10^8^ CFU/mL bacterial suspension was injected to establish the *G. mellonella* infection model (*n* = 6). One hour post-infection, 5 µL of the phage Henu2_3 suspension was injected at MOIs of 100, 10, 1 and 0.1. After 24 h of incubation at 37 °C in the dark, the weight of *G. mellonella* larvae was recorded, and bacterial loads were quantified and expressed as CFU/g.

### Antibacterial effect of phage Henu2_3 in mouse infection model

An experimental mouse model of pneumonia was established by intraperitoneal injection of *K. pneumoniae* Kp1049. Briefly, the bacterial culture was grown in LB medium to logarithmic phase (1 × 10^8^ CFU/mL). The bacterial cultures were then centrifuged (8,500 × g, 5 min) and washed twice with saline. The bacterial suspension was adjusted to a concentration of 1 × 10^11^ CFU/mL for survival rate experiments and 1 × 10^10^ CFU/mL for bacterial load quantification. A volume of 100 µL of the bacterial suspension was injected into the peritoneal cavity of mice to establish a bacteremia infection model. Phage solutions were prepared at different MOI ratios (0.01–100) and administered intraperitoneally 1 h post-bacterial infection, with saline injection serving as the negative control. For the survival rate assessment, thirty female C57BL/6 N mice were intraperitoneally injected with the bacterial suspension and divided into six groups (*n* = 5 per group). The survival rates of the infected mice were monitored and recorded over a 7-day period. For bacterial load analysis, twelve female C57BL/6 N mice were intraperitoneally injected with the bacterial suspension and divided into three groups (*n* = 4 per group). After 48 h of treatment, the mice were euthanized, and their organs (heart, liver, spleen, lung, and kidney) were collected. Bacterial loads in the organs were quantified using CFU assays on 1.5% LB agar plates to evaluate the therapeutic efficacy of phage Henu2_3 in the pneumonia mouse model.

## Results

### Isolation and morphology of phage Henu2_3

**Fig. 1:**
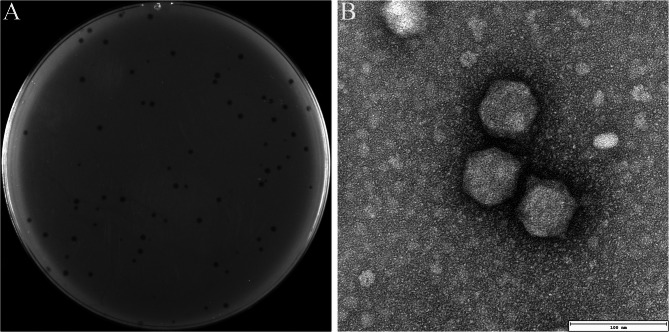
Morphological characterization of phage Henu2_3. **A** Plaque morphology of phage Henu2_3 on *K. pneumoniae* Kp1049 host lawn, demonstrating clear lytic zones. **B** TEM image of phage Henu2_3 virions, revealing structural details. Scale bar: 100 nm

The phage of Henu2_3 was isolated from hospital sewage from The First Affiliated Hospital of Henan University using the clinical isolate *K. pneumoniae* Kp1049 as the host. The purified phage Henu2_3 was applied on the lawn of *K. pneumoniae* Kp1049, resulting in the formation of clear and well-defined plaques after 12 h of incubation at 37℃ (Fig. 1A). TEM analysis revealed that phage Henu2_3 possessed an icosahedral head with an average diameter of 63.71 ± 0.87 nm and a short tail (Fig. 1B).

### The host range of phage Henu2_3

The host range of phages is critically important for clinical applications. To evaluate the host range of phage Henu2_3, its infectivity was tested against fifteen clinical isolates of *K. pneumoniae* and other gram-negative bacteria. The capsular types of *K. pneumoniae* strains were determined through *wzi* gene sequencing (Brisse et al. 2013). The results revealed that phage Henu2_3 infected all tested K1-type *K. pneumoniae* strains but showed no infectivity against other capsular types of *K. pneumoniae*, *E. coli*, *A. baumannii*, or *P. aeruginosa* (Table 1). These findings demonstrated that Henu2_3 is a K1 type-specific *Klebsiella* phage with a narrow and strict host range.

**Table 1:** Host range analysis of phage Henu2_3 against clinical isolates

Species	Strains	Capsular type	Susceptibility	Origin
*K. pneumoniae*	Kp1049	K1	+	The First Affiliated Hospital of Henan University
	Kp0311	K1	+	The First Affiliated Hospital of Henan University
	Kp0822	K1	+	The First Affiliated Hospital of Henan University
	Kp413	K1	+	Henan Provincial People’s Hospital
	Kp120806	K1	+	Henan Provincial People’s Hospital
	Kp120809	K1	+	Henan Provincial People’s Hospital
	Kp408	K2	−	Henan Provincial People’s Hospital
	Kp1616	K2	−	The First Affiliated Hospital of Henan University
	Kp0524	K2	−	The First Affiliated Hospital of Henan University
	Kp2869	K2	−	The First Affiliated Hospital of Henan University
	Kp120801	K19	−	Henan Provincial People’s Hospital
	Kp120804	K62	−	Henan Provincial People’s Hospital
	Kp120819	K63	−	Henan Provincial People’s Hospital
	Kp2640	K28	−	The First Affiliated Hospital of Henan University
	Kp120803	K14,K64	−	Henan Provincial People’s Hospital
*E. coli*	BW25113		−	Shanghai Microbial Collection Center
*A. baumannii*	Ab2055		−	The First Affiliated Hospital of Henan University
*P. aeruginosa*	Pa3046		−	The First Affiliated Hospital of Henan University

### Biological characterization of phage Henu2_3

**Fig. 2:**
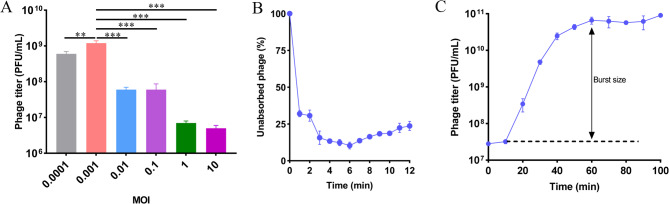
Lytic characterization of phage Henu2_3. **A** Determination of the optimal MOI for phage Henu2_3, defined as the phage-to-host ratio yielding the highest titer. **B** Adsorption curve of the phage Henu2_3. The phage Henu2_3 was mixed with *K. pneumoniae* Kp1049 to reach the optimal MOI of 0.001. While the mixture was incubated with shaking, samples were taken every minute for PFU determined. **C** One-step growth curve of phage Henu2_3, depicting the latent period, burst size, and rise phase. Data represent means ± SD (*n* = 3), statistical analyses were conducted using Student’s *t*-test for comparisons between groups. ***p < 0.001, **p < 0.01, *p < 0.05

To determine the optimal MOI for phage Henu2_3, we conducted co-culture experiments with exponentially growing *K. pneumoniae* Kp1049 cells at varying phage-to-bacteria ratios. Phage Henu2_3 exhibited peak progeny production at an MOI of 0.001, indicating that this ratio maximizes viral yield (Fig. 2A). This low MOI suggests that a single phage per 1,000 bacterial cells is sufficient for efficient replication, making it ideal for large-scale phage propagation and therapeutic applications where resource efficiency is critical. Quantitative adsorption kinetics demonstrated that phage Henu2_3 achieved 90% host binding within 6 min of exposure, demonstrating that high host affinity and potential therapeutic advantage (Fig. 2B). To characterize the replication dynamics of phage Henu2_3, we conducted a comprehensive one-step growth analysis under optimized conditions. The exponentially growing *K. pneumoniae* Kp1049 was mixed with phage Henu2_3 at an MOI of 0.001. The mixture was incubated at 37℃ for 10 min to allow sufficient adsorption of the phage to the bacteria. The bacteria were collected and washed twice with PBS and resuspended in fresh LB medium. Phage titers were measured at 10 min intervals over a 100 min period. One-step growth curve was drawn by taking infection time as the abscissa and phage titer as the ordinate (Fig. 2C). The results showed that the latent time was 10 min and the burst size was 215 PFU/infected cells.

### The stability of phage Henu2_3

**Fig. 3:**
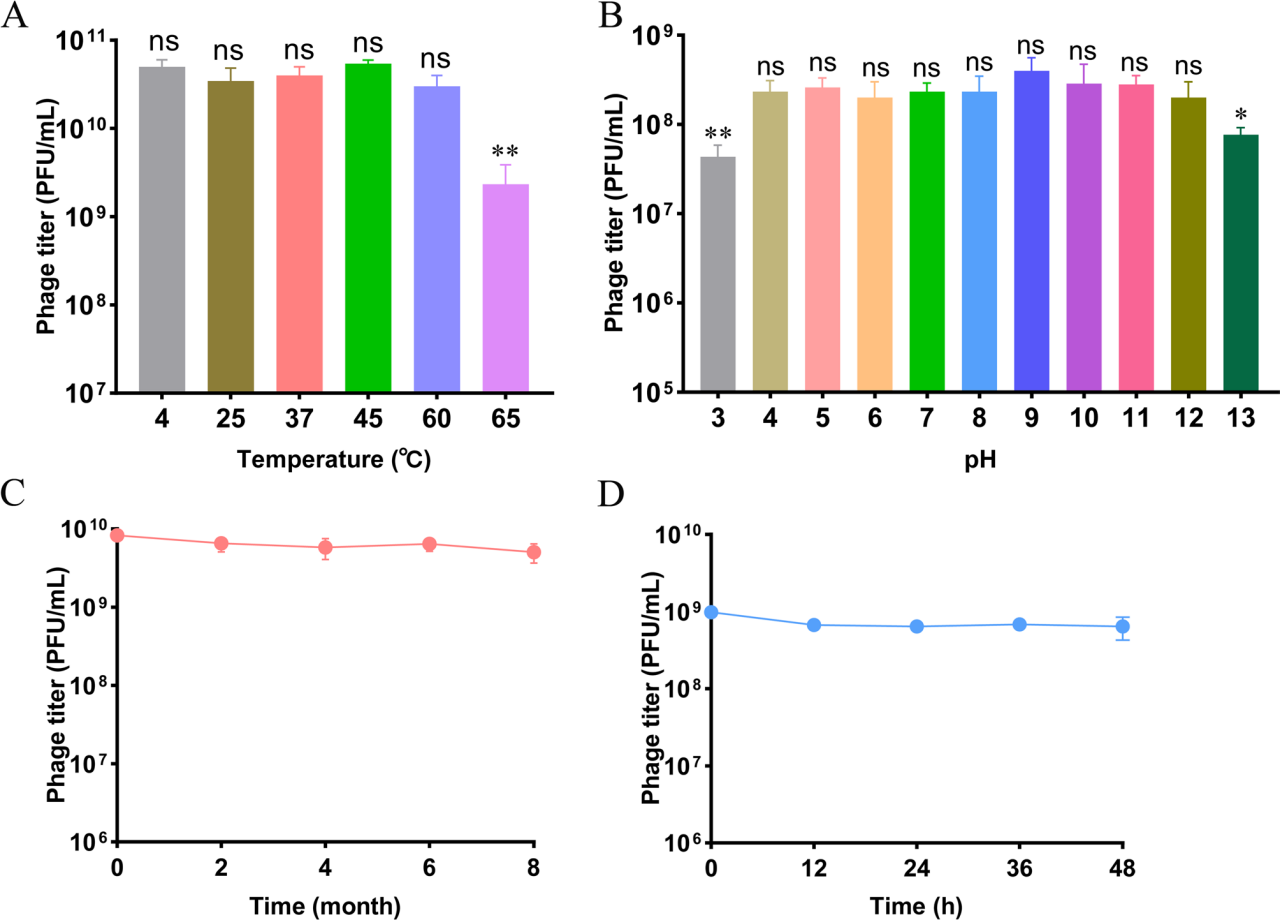
Stability profile of phage Henu2_3 under various conditions. **A** Thermal stability of phage Henu2_3 incubated at different temperatures (4–65℃) for 1 h. **B** pH stability of phage Henu2_3 exposed to buffers ranging from pH 3 to 13. **C** Long-term storage stability of phage Henu2_3 at 4℃ over 8 months. **D** Serum stability of phage Henu2_3, demonstrating maintained lytic activity in serum for 48 h. Data represent mean ± SD (*n* = 3), statistical analysis was performed by one-way analysis of variance following a Dunnett’s multiple comparisons test. **p < 0.01, *p < 0.05, ns, not significant

To evaluate the potential of developing phage Henu2_3 into a clinically viable formulation, its stability under varying thermal conditions, pH levels, and long-term storage was systematically assessed. Phage Henu2_3 demonstrated high stability across a range of temperatures (4℃, 25℃, 37℃, 45℃, 60℃ and 65℃) over a 1 h period (Fig. 3A). Phage Henu2_3 maintained high activity across a broad pH range (pH 4–12), but its titer decreased sigbificantly at pH 3 and pH 13 (Fig. 3B). Furthermore, its long-term stability was evaluated by storing the phage at 4℃ and pH = 7, with monthly monitoring over an 8-month period. The results revealed no significant reduction (*P* = 0.1397) in phage titer throughout the 8 months, demonstrating excellent long-term stability of phage Henu2_3 (Fig. 3C). Subsequently, we evaluated the infection stability of phage Henu2_3 in serum. The results demonstrated that co-incubation with serum for 48 h did not compromise the lytic activity of phage Henu2_3 (Fig. 3D). These results demonstrated that phage Henu2_3 maintains stable lytic activity across various environmental conditions and remains suitable for long-term storage at 4℃.

### Genomic characterization and bioinformatic analysis of phage Henu2_3

**Fig. 4:**
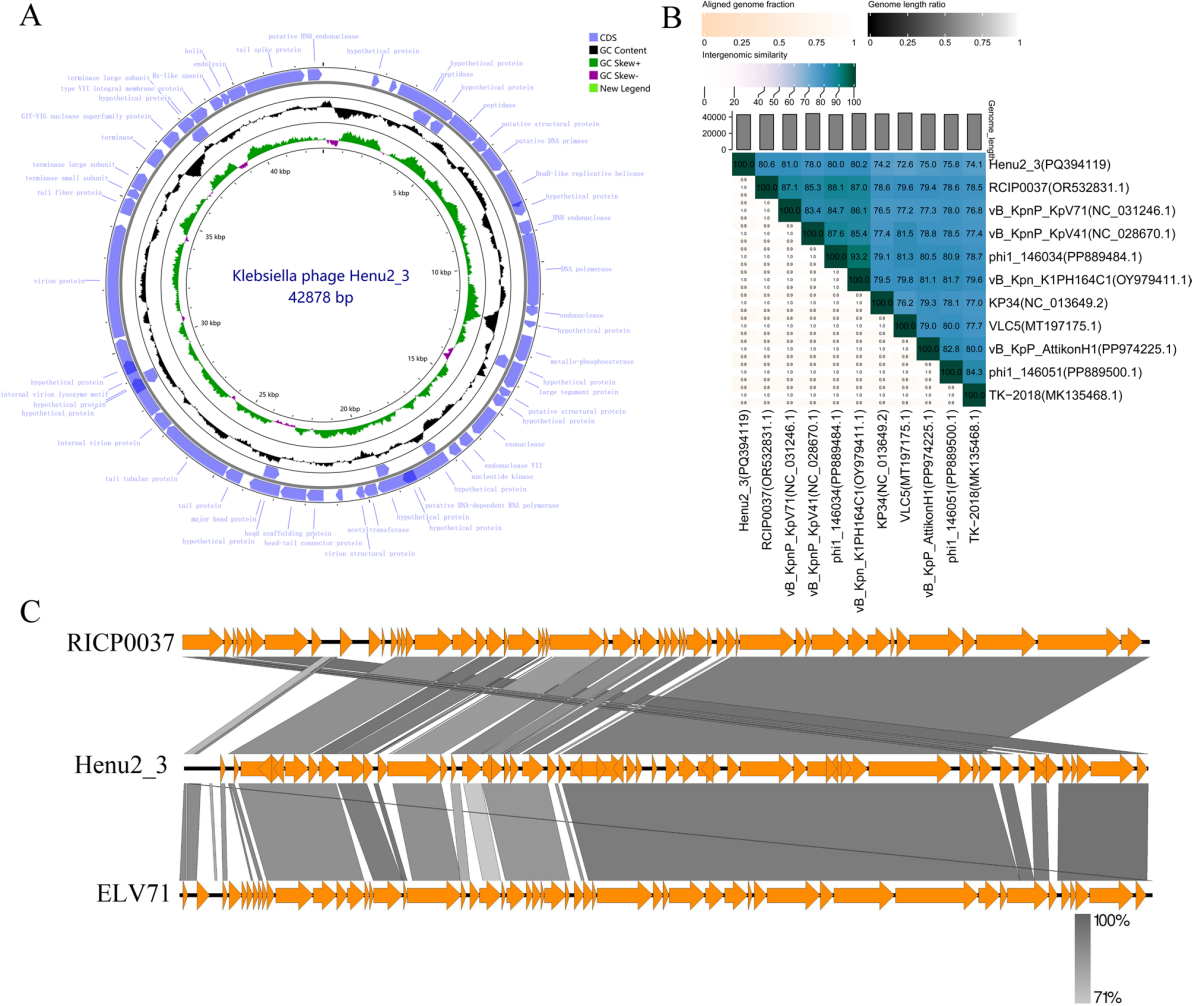
Comparative genomic analysis of phage Henu2_3. **A** The genome map of Henu2_3, annotated with predicted ORFs. **B** Heatmap of pairwise genome similarity percentages among phage Henu2_3 and related phages. The values were calculated using VIDIRIC software. The horizontal and vertical coordinates indicate the corresponding phage and Genebank number. **C** Multiple genome alignments of phage Henu2_3 with reference phages RICP0037 and ELV71, showing regions of local colinearity (locally collinear blocks) and sequence divergence

**Fig. 5:**
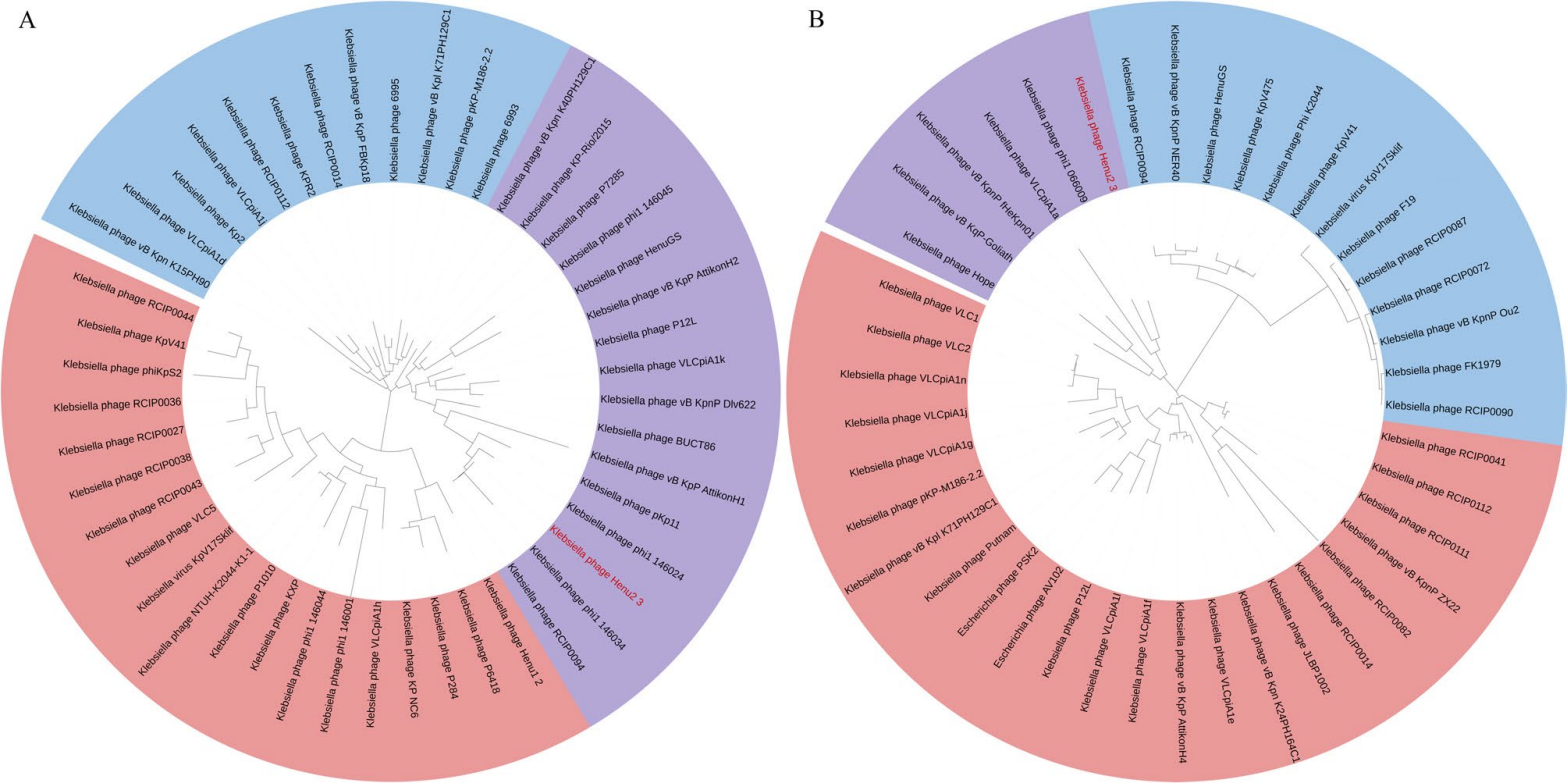
Phylogenetic analysis of phage Henu2_3. Phylogenetic trees were constructed using the neighbor-joining method in MEGA7 based on amino acid sequences of (**A**) major capsid protein and (**B**) DNA polymerase, with bootstrap values calculated from 1000 replicates. Reference sequences were obtained from NCBI. Henu2_3 is highlighted in red, while phages belonging to co-evolutionary clusters are color-coded

To investigate the genomic characteristics and potential encoded proteins of phage Henu2_3, we performed whole-genome sequencing of this phage. The results showed that phage Henu2_3 possessed a linear double-stranded DNA (dsDNA) genome of 42,878 bp (GenBank: PQ394119.1) with a G + C content of 53.97% (Fig. 4A). The genomic DNA of phage Henu2_3 was susceptible to cleavage by multiple restriction endonucleases, it could be cleaved by *Bam*HI (with only one restriction site) into two fragments, further confirming its linear genome structure (Figure S1). According to Softberry and BLASTp analysis results, a total of 54 open reading frames (ORFs) were predicted in the phage Henu2_3 complete genome (Table 2). In addition, no virulence, antimicrobial resistance and tRNA genes were annotated in the phage Henu2_3 gemome. The complete genome sequence alignment of phage Henu2_3 with the complete genome sequences of other phages in the NCBI data conducted using the VIRIDIC tool showed that Henu2_3 shared the highest homology with RCIP0037 (GenBank: OR532831.1), vB_KpnP_KpV71 (GenBank: NC_031246.1), vB_KpnP_KpV41 (GenBank: NC_028670.1), phi1_146034 (GenBank: PP889484.1) and vB_Kpn_K1PH164C1 (GenBank: OY979411.1) (Fig. 4B). Comparative genomic analysis using Easyfig revealed that phage Henu2_3 shares significant sequence similarity with phage RCIP0037 and vB_KpnP_KpV71, particularly within the gp23-gp43 genomic region (Fig. 4C). The phylogenetic analysis was conducted based on the the conserved amino acid sequences of the DNA polymerase (Gp12) and major capsid protein (Gp32) encoded by phage Henu2_3, along with homologous sequences from related phages. Comparative phylogenetic analysis demonstrated that phage Henu2_3 shares closer genetic distance with Klebsiella phages, phi1_146034 and RCIP0094, when examining DNA polymerase sequences, whereas major capsid protein sequence alignment showed stronger evolutionary relationship with *Klebsiella* phage phi1_066009 (Fig. 5A and B).

**Table 2:** Features of the open reading frames (ORFs) of phage Henu2_3

ORF	Strand	Start	Stop	Length (AA)	Predicted Protein Function	Best-match BLASTp Result	Query cover	E-values	Identity	Accession	MW (kDa)
1	+	1621	1845	74	Hypothetical protein	*Klebsiella* phage vB_Kpn_HF0522	100%	3e−43	97.30%	XBS48880.1	8.36
2	+	2226	2444	72	GABARAP domain of GABA-receptor-associated protein	Caudoviricetes sp.	100%	2e−31	76.39%	DAV54610.1	7.86
3	+	2529	4496	655	Peptidase	*Klebsiella* phage pKP-M212−2.1	98%	0	87.93%	WJZ69626.1	73.73
4	−	3288	3905	205	No fit						22.48
5	−	3996	4427	143	No fit						15.67
6	+	4496	5542	348	Peptidase	*Klebsiella* phage BUCT631	100%	0	96.55%	WAK45682.1	39.03
7	+	5545	6006	153	Putative structural protein	*Klebsiella* phage vB_KpnP_fHeKpn01	100%	2e−52	58.44%	QFG06561.1	17.43
8	+	6006	6797	263	Putative DNA primase	*Klebsiella* phage vB_Kp_XP4	100%	0	98.48%	XAG93544.1	29.33
9	+	6871	8127	418	DnaB-like replicative helicase	Caudoviricetes sp.	99%	0	99.76%	DAT39320.1	46.75
10	+	7982	8428	148	Hypothetical protein	*Klebsiella* phage KMI6	97%	1e−67	72.60%	QEG10134.1	16.63
11	+	8627	9082	151	HNH endonuclease	*Klebsiella* phage vB_KpP_AttikonH1	100%	2e−104	96.69%	XFD46794.1	16.67
12	+	9051	11,429	792	DNA polymerase	*Klebsiella* phage VLCpiA1f	100%	0	93.59%	UVX31444.1	90.95
13	+	11,407	11,724	105	Endonuclease	Caudoviricetes sp.	100%	2e−61	87.62%	DAH76656.1	11.6
14	+	11,891	12,112	73	Hypothetical protein	*Klebsiella* phage KpV475	100%	2e−43	100%	YP_009280686.1	8.08
15	+	12,377	13,249	290	Metallo-phosphoesterase	*Klebsiella* phage VLCpiA1f	100%	0	98.28%	UVX31442.1	32.43
16	−	13,211	13,660	149	Hypothetical protein	*Klebsiella* phage Henu1_2	91%	3e−57	77.94%	XEN42093.1	16.69
17	+	13,304	14,182	292	Large tegument protein	*Klebsiella* phage Henu1_1	100%	0	95.89%	XEN42151.1	30.82
18	+	14,235	14,513	92	Putative structural protein	*Klebsiella* phage vB_Kp_XP4	100%	5e−54	92.39%	XAG93555.1	10.73
19	+	14,513	14,884	123	Hypothetical protein	*Klebsiella* phage RCIP0099	100%	1e−78	99.19%	WPJ55983.1	13.19
20	+	15,045	16,013	322	Exonuclease	*Klebsiella* phage F19	100%	0	97.20%	YP_009006045.1	36.54
21	+	16,164	16,586	140	Endonuclease VII	*Klebsiella* phage KpIITR008	100%	1e−81	95%	XAQ48560.1	15.65
22	+	16,670	17,041	123	Nucleotide kinase	*Klebsiella* phage vB_KpnP_SU503	100%	1e−75	88.62%	YP_009199915.1	14.06
23	+	17,186	19,654	822	Putative DNA-dependent RNA polymerase	*Klebsiella* phage VLC5	100%	0	98.54%	QIW86407.1	92.91
24	−	17,260	17,685	141	Hypothetical protein	*Klebsiella* phage Henu1_2	100%	2e−89	95.74%	XEN42102.1	15.65
25	+	18,360	18,788	142	Hypothetical protein	*Klebsiella* phage Henu1_2	100%	2e−46	64.79%	XEN42103.1	16.29
26	−	19,076	19,528	150	No fit						17.04
27	+	19,678	20,118	146	Acetyltransferase	*Klebsiella* phage vB_KpP_AttikonH1	100%	1e−96	93.84%	XFD46814.1	16.38
28	+	20,115	20,378	87	Virion structural protein	*Klebsiella* phage vB_KpP_AttikonH1	100%	3e−50	98.85%	XFD46815.1	8.74
29	+	20,812	21,039	75	Hypothetical protein	*Klebsiella* phage Henu1_2	100%	3e−44	100%	XEN42107.1	7.84
30	+	21,429	21,983	184	Head-tail connector protein	*Klebsiella* phage Henu1_2	100%	1e−130	100%	XEN42108.1	20.09
31	+	21,998	22,840	280	Head scaffolding protein	*Klebsiella* phage vB_Kpn_HF0522	100%	0	99.29%	XBS48912.1	30.1
32	+	22,866	23,885	339	Major capsid protein	*Klebsiella* phage phi1_146024	99%	0	100%	XCO39008.1	37.7
33	−	22,989	23,528	179	Hypothetical protein	Caudoviricetes sp.	100%	4e−96	83.24%	DAH76667.1	19.59
34	+	24,166	24,726	186	Tail protein	*Klebsiella* phage KpV71	100%	3e−134	100%	YP_009302744.1	21.35
35	+	24,736	27,108	790	Tail tubular protein	*Klebsiella* phage RCIP0072	100%	0	98.61%	WPJ54186.1	86.39
36	+	27,110	27,697	195	Internal virion protein	*Klebsiella* phage VLCpiA1d	100%	3e−136	100%	UVX31813.1	20.48
37	+	27,715	30,399	894	Internal virion lysozyme motif	*Klebsiella* phage KpV71	100%	0	99.44%	YP_009302747.1	97.41
38	+	28,556	29,020	154	Hypothetical protein	*Klebsiella* phage Henu1_2	100%	2e−101	98.70%	XEN42115.1	17.69
39	−	28,599	29,078	159	Hypothetical protein	*Klebsiella* phage Henu1_2	100%	8e−105	99.37%	XEN42116.1	17.28
40	+	29,219	29,656	145	Hypothetical protein	*Klebsiella* phage Henu1_2	100%	6e−91	100%	XEN42118.1	16.28
41	+	30,450	34,148	1232	Virion protein	Caudoviricetes sp.	100%	0	98.46%	DAT39314.1	134.19
42	+	34,495	35,073	192	Tail fiber protein	Caudoviricetes sp.	100%	8e−127	97.40%	DAT39315.1	19.8
43	+	35,085	35,387	100	Terminase small subunit	*Klebsiella* phage vB_KpnP_KpV74	100%	2e−62	100%	YP_009789288.1	11.21
44	+	35,387	35,959	190	Terminase large subunit	Acinetobacter phage vB_AbaA_LLY	100%	4e−135	98.95%	WEV89140.1	21.19
45	+	36,303	36,917	204	Terminase	Caudoviricetes sp.	100%	7e−149	100%	DAT39318.1	23.28
46	+	37,126	37,677	183	GIY-YIG nuclease superfamily protein	Caudoviricetes sp.	100%	2e−133	98.91%	DAT39351.1	21.04
47	+	37,822	38,340	172	Terminase large subunit	*Klebsiella* phage P284	100%	1e−117	97.67%	XDJ01860.1	19.52
48	−	37,879	38,448	189	Hypothetical protein	*Klebsiella* phage Henu1_2	82%	2e−91	86.45%	XEN42124.1	21.26
49	+	38,340	38,882	180	Type VII integral membrane protein	Pectobacterium phage Peat1	100%	3e−12	38.50%	YP_009224684.1	17.74
50	+	39,082	39,486	134	Rz-like spanin	*Klebsiella* phage KpV41	100%	3e−86	98.51%	YP_009188794.1	14.04
51	+	39,479	39,730	83	Holin	Acinetobacter phage vB_AbaA_LLY	100%	2e−51	98.80%	WEV89146.1	9.15
52	+	39,714	40,322	202	Endolysin	*Klebsiella* phage P284	100%	3e−144	98.02%	XDJ01910.1	21.91
53	+	40,323	42,278	651	Tail spike protein	*Klebsiella* phage vB_Kpn_K1PH164C1	100%	0	98.92%	CAK6604185.1	69.94
54	+	42,384	42,845	153	Putative HNH endonuclease	*Klebsiella* phage vB_KpnP_Bp5	99%	1e−108	98.68%	QDJ96113.1	17.79

### Evaluation of the antibacterial and anti-biofilm effects of phage Henu2_3

**Fig. 6:**
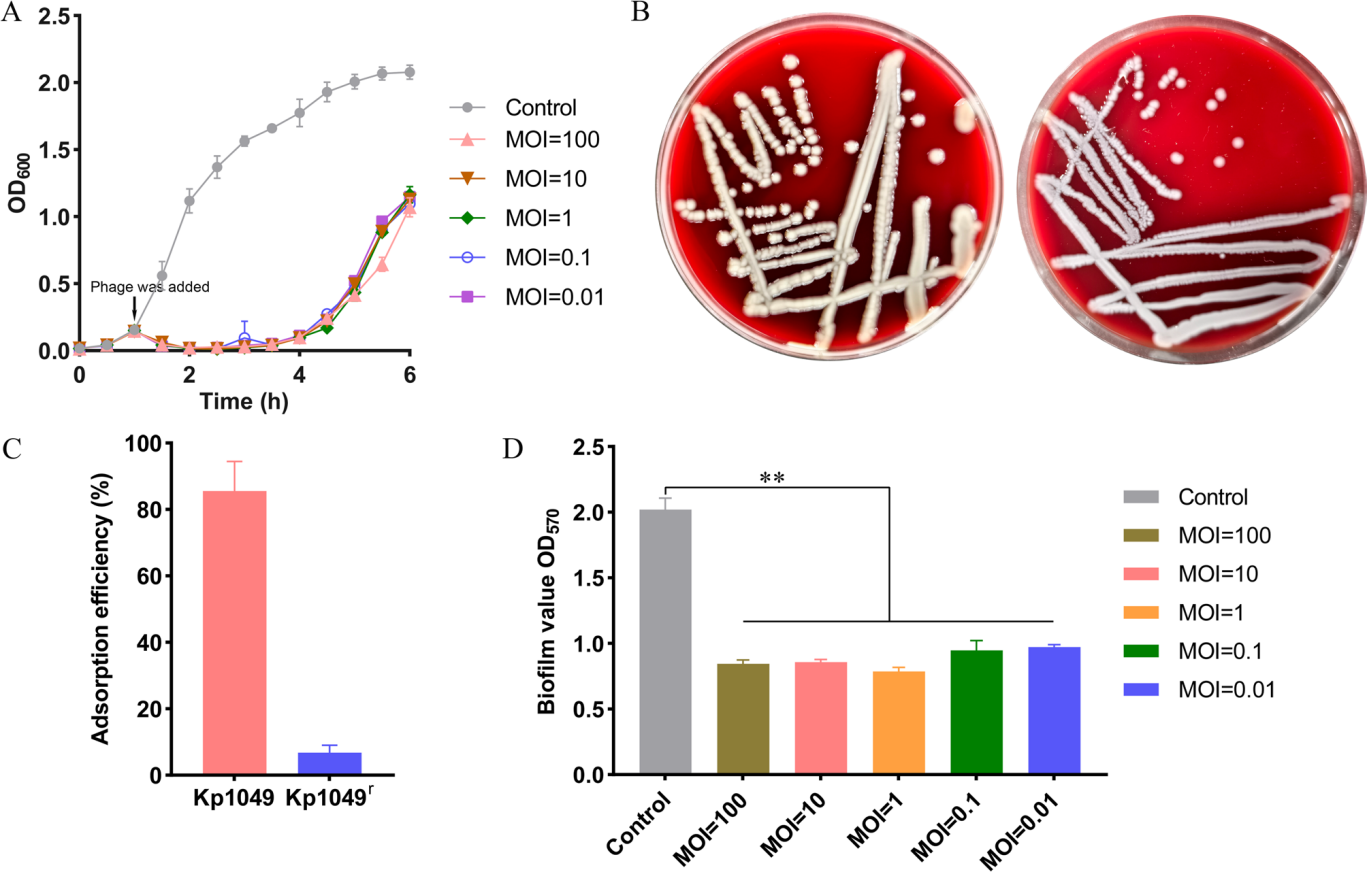
Antimicrobial and antibiofilm activity of phage Henu2_3 in vitro. **A** Growth inhibition of *K. pneumoniae* Kp1049 by phage Henu2_3 at MOI of 0.01, 0.1, 1, 10, 100, monitored by optical density (OD_600_) over time. **B** The colony morphology of *K. pneumoniae* Kp1049 and phage-resistant strains on blood agar plates. **C** Detection of the adsorption rate of phage Henu2_3 to *K. pneumoniae* Kp1049 and phage-resistant strains (Kp1049^r^). **D** Quantitative assessment of biofilm disruption, showing reduction in mature *K. pneumoniae* Kp1049 biofilms after phage Henu2_3 treatment (MOI = 0.01, 0.1, 1, 10, 100) as measured by crystal violet staining (OD_570_). Data represent means ± SD from three independent experiments (**p < 0.001, Student’s *t*-test)

To evaluate the antibacterial activity of phage Henu2_3, we monitored growth curves of host bacteria incubated with different MOIs (0.01, 0.1, 1, 10, 100). Results demonstrated complete bacterial growth inhibition within the first 4 h (Fig. 6A). However, bacterial cultures gradually entered logarithmic phase after 4 h, exhibiting rapid proliferation. The rapidly growing bacterial populations were isolated and cultured on solid agar plates. After 12 h of incubation, we observed significant morphological alterations in phage-treated colonies compared to the control group (Fig. 6B). The morphologically altered strain developed resistance to the phage and significantly impaired phage adsorption to the host bacterial surface (Figure S2 and Fig. 6C). Biofilms formed by bacteria under harsh environmental conditions are notoriously resistant to antibiotic eradication and represent a major cause of persistent infections. In contrast, phages demonstrate superior biofilm-clearing capabilities compared to antibiotics (Abedon 2015; Chegini et al. 2020). We therefore evaluated the biofilm eradication capacity of phage Henu2_3. Our results demonstrated that phage Henu2_3 significantly reduced mature biofilm biomass across all tested MOIs following 24 h incubation (Fig. 6D).

### Antibacterial efficacy of phage Henu2_3 in animal infection models

**Fig. 7:**
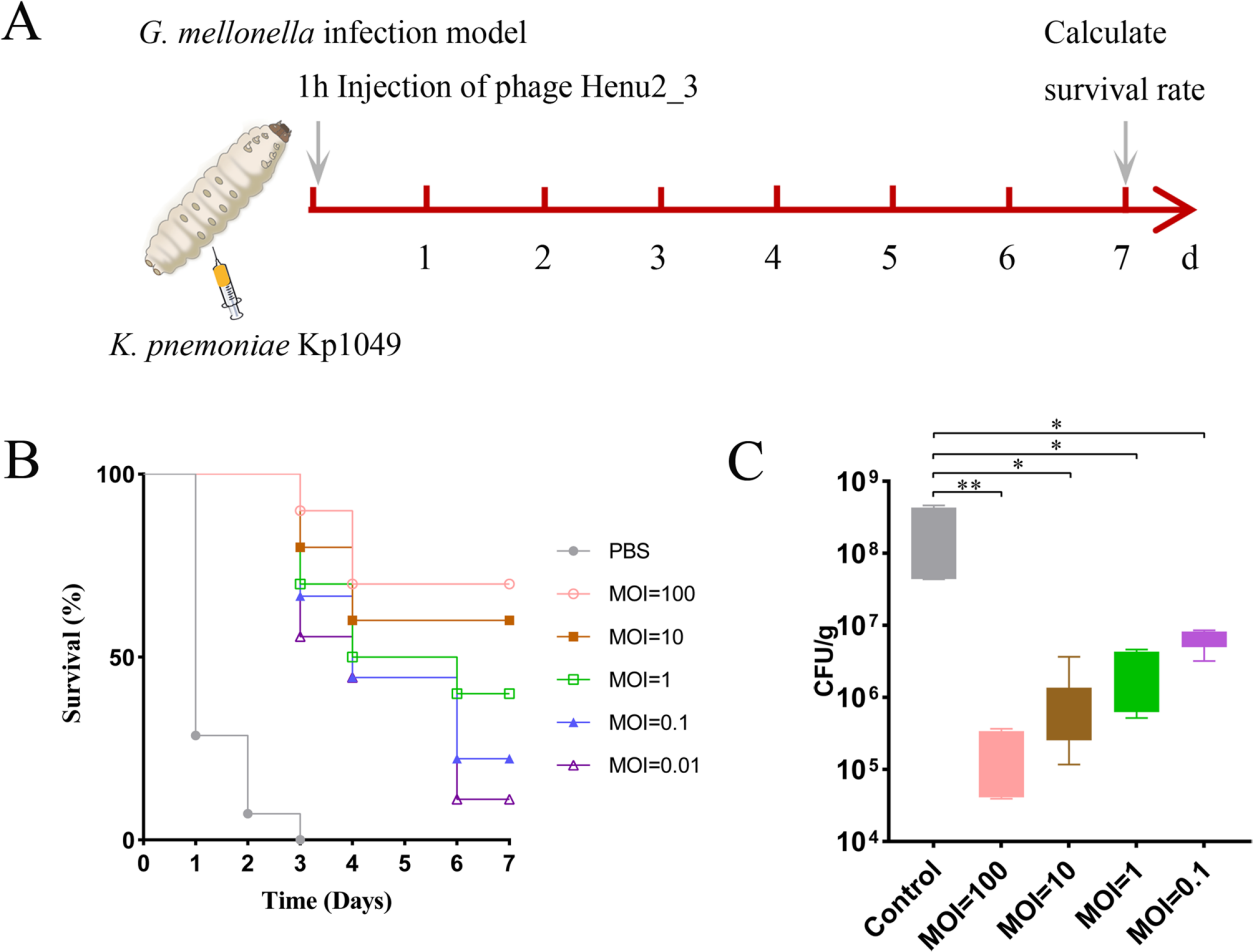
Therapeutic efficacy of phage Henu2_3 against *K. pneumoniae* Kp1049 in *G. mellonella* infection models. **A** Experimental design schematic showing time points for bacterial infection, phage administration, and outcome assessments. **B** The survival curves of *G. mellonella* larvae (*n* = 10 per group) following infection with *K. pneumoniae* Kp1049 and treatment with phage Henu2_3 at varying MOIs (0.01, 0.1, 1, 10, 100). **C** Quantitative bacterial burden in larvae (*n* = 6 per group) 24 h post-treatment, determined by serial dilution plating (log10 CFU/g). Statistical significance was determined by Student’s *t*-test (*p < 0.05, **p < 0.01, vs. infected control)

**Fig. 8:**
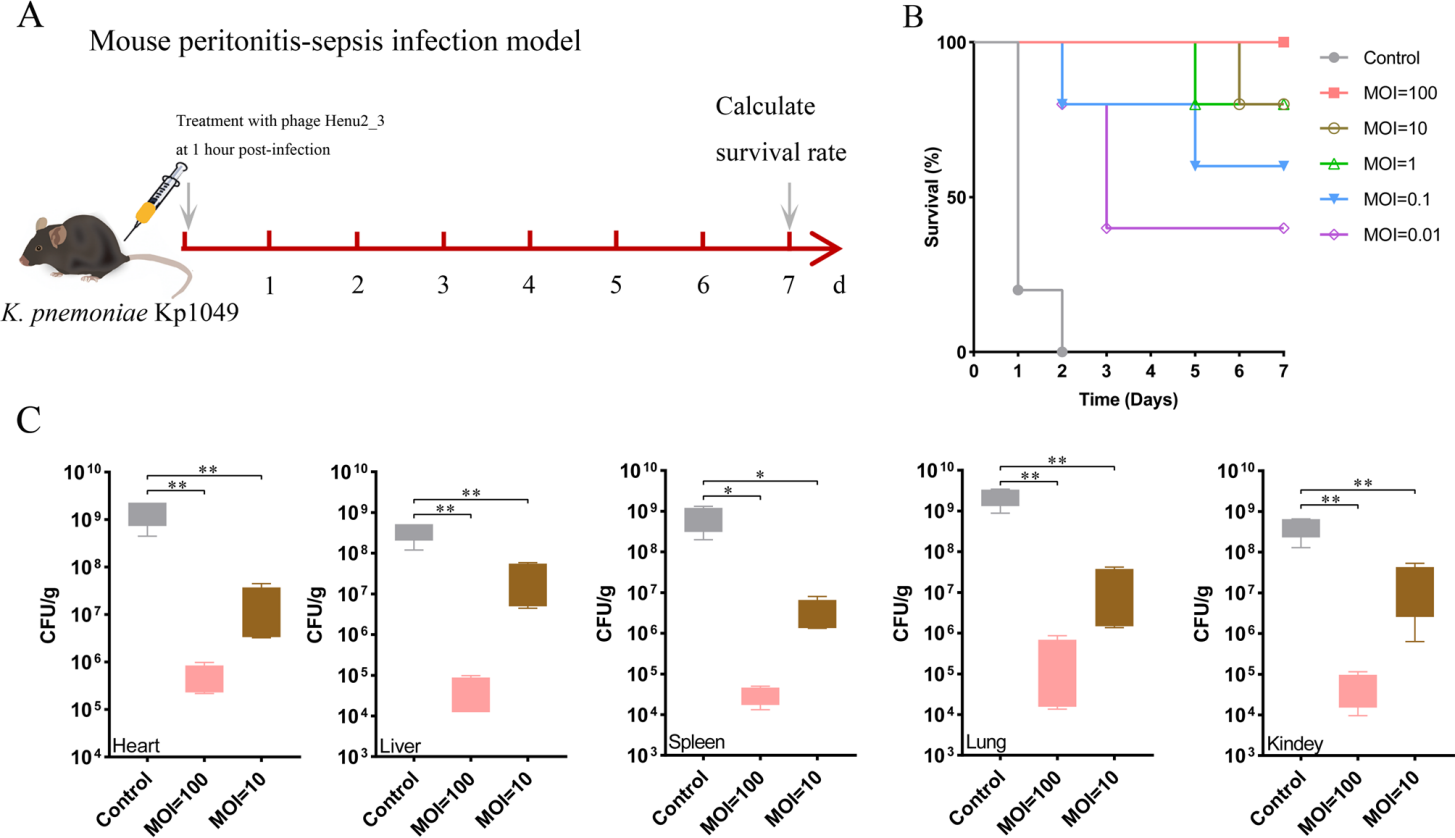
Therapeutic efficacy of phage Henu2_3 in murine *K. pneumoniae* Kp1049 infection models. **A** Experimental timeline showing infection and phage treatment administration at various MOIs. **B** Survival kinetics of C57BL/6 N mice (*n* = 5 per group) following *K. pneumoniae* Kp1049 challenge and Henu2_3 therapy at MOIs ranging from 0.01 to 100, monitored for 7 days. **C** Quantitative bacterial burden in major organs (heart, liver, spleen, lungs and kindey) of bacteremic mice (*n* = 4 per group) 48 h post-treatment with phage Henu2_3 at different MOIs. Statistical significance was determined by Student’s *t*-test (*p < 0.05, **p < 0.01, ***p < 0.001 vs. untreated controls)

The above experimental results demonstrated that the antibacterial stability of Henu2_3 in vitro, suggesting its potential for clinical applications. To further evaluate the clinical prospects of phage Henu2_3, we subsequently assessed its antimicrobial efficacy using two animal infection models. First, we employed a simple *G. mellonella* larvae infection model to investigate the effects of phage Henu2_3 on both host survival rates and bacterial loads in vivo (Fig. 7A). The results showed that at lethal infection dose, phage Henu2_3 treatment at different MOIs could significantly improve the survival rates of *G. mellonella* larvae (Fig. 7B). Subsequently, under sublethal infection conditions, we observed that phage Henu2_3 treatment markedly reduced the bacterial burden in the *G. mellonella* larvae (Fig. 7C). Subsequently, we intraperitoneally injected *K. pneumoniae* to establish a sepsis mouse model for evaluating the therapeutic efficacy of phage Henu2_3 (Fig. 8A). Our results demonstrated that phage Henu2_3 could significantly improve the survival rate of septic mice. Remarkably, in the high MOI treatment group, the survival rate reached up to 100% (Fig. 8B). We next examined bacterial loads in various organs of sublethally infected mice following 48 h phage therapy. The results demonstrated that phage Henu2_3 treatment significantly reduced bacterial burdens in all examined organs (heart, liver, spleen, lungs, and kidneys) (Fig. 8C). Importantly, we observed a clear dose-dependent therapeutic effect - higher administered phage titers correlated with greater reductions in bacterial colonization.

## Discussion

With the widespread emergence of bacterial resistance and the extreme difficulty in developing new antibiotics, phage therapy has become a promising approach for bacterial treatment in the post-antibiotic era (Luong et al. 2020). The global rise of antibiotic resistance in *K. pneumoniae* poses a critical challenge to the medical community worldwide. However, of greater concern is the global spread of a hypervirulent and MDR strain of *K. pneumoniae*, which has now been reported in at least 16 countries (Zheng et al. 2025). Bacteriophage therapy represents a promising alternative therapeutic approach for combating antibiotic-resistant *K. pneumoniae* strains (Abbas et al. 2025). The bacteriophage Henu2_3, isolated from sewage samples at The First Affiliated Hospital of Henan University, demonstrates effective lytic activity against hvKp. Biological characteristics tests showed that phage Henu2_3 has good tolerance to temperature and pH. The exceptionally low optimal MOI, short incubation period, and association with large outbreaks collectively demonstrated that Henu2_3 possesses remarkable proliferation efficiency and potent lytic activity. Phages capable of rapidly recognizing and lysing bacteria will play a significant role in clinical applications. Quick bacterial targeting, which is crucial for acute infection control. The bacteriophage Henu2_3 exhibited a burst size of 215 PFU/per infected cell at the optimal MOI, indicated that it was a virulent phage. Furthermore, host range analysis revealed exceptionally high specificity of this phage. These characteristics suggested that phage Henu2_3 represents a promising candidate strain for phage therapy applications.

Bacteriophages exhibit remarkable morphological diversity. Based on their structural characteristics, phage particles can be classified into tailed phages, tail less phages, enveloped phages, and filamentous phages (Dion et al. 2020). Additionally, depending on the type of genetic material, phages can be categorized as dsDNA, single-stranded DNA (ssDNA), double-stranded RNA (dsRNA), or single-stranded RNA (ssRNA) phages (Dion et al. 2020). According to morphological classification, phage Henu2_3 belongs to the family of short tail phages, while based on nucleic acid type, phage Henu2_3 is classified as a dsDNA phage. Phages KP34 and SU552A exhibit significant sequence homology with phage Henu2_3 and are taxonomically classified within the *Autographivirales* order, *Autoscriptoviridae* family, *Slopekvirinae* subfamily, *and Drulisvirus* genus. The morphological characteristics of phage Henu2_3, as observed by TEM, confirmed its taxonomic classification within the *Drulisvirus* genus.

Whole genome sequencing of phage Henu2_3 generated 7,652,390 total reads with a Q30 quality-score of 92.87%. The *de novo* assembly produced a single linear contig spanning 42,878 bp with a G + C content of 53.97%, which is similar to most *Klebsiella* phages. Phage Henu2_3 DNA was sensitive to digestion by a variety of restriction endonucleases (*Bam*HI, *Eco*RI, *Hin*dIII and *Xho*I) incicated that it was unmodified dsDNA. Notably, only a single *Bam*HI restriction site was identified in the genome of phage Henu2_3 (Figure S1A). However, digestion with *Bam*HI yielded two distinct DNA fragments (35, 809 bp and 7069 bp), confirmed that the Henu2_3 genome exists in a linear form (Figure S1B). The genome type of bacteriophages (linear or circular) does not affect their ability to rapidly lyse host bacteria, and both can be applied clinically under appropriate infection conditions. Genomic analysis identified 54 ORFs in phage Henu2_3, with functional annotation revealing that 38 ORFs encode proteins of known function while 12 remain functionally uncharacterized. The HNH (His-Asn-His) domain represents a ubiquitous family of compact nucleic acid-binding proteins, universally distributed across all biological kingdoms and typically associated with endonuclease activity. Notably, HNH domains are particularly prevalent in phage-encoded proteins. The HNH protein from phage P1 enhances bacterial lethality during lytic induction by DNA damage through its DNA-binding and nuclease activities (Ronayne et al. 2016). Among the functionally annotated proteins encoded by phage Henu2_3, two ORFs (gp11 and gp54) were predicted to encode HNH endonucleases. The tail fiber protein and tail spike protein of phages may encode polysaccharide depolymerases that hydrolyze the capsule of *K. pneumoniae*, resulting in halo formation around phage plaques. However, although phage Henu2_3 genomes also encode tail fiber and tail spike proteins, no halo was observed around their plaques. Phages exhibit remarkable diversity, and similarly, the proteins they encode demonstrate extensive heterogeneity. Consequently, investigating the functions of phage-encoded proteins will facilitate their clinical applications.

Phage Henu2_3 demonstrates potent lytic activity against both rapidly dividing bacterial cells and established biofilms. The anti-biofilm efficacy of phage Henu2_3 was particularly notable against mature biofilms, with all tested MOIs achieving statistically significant biomass reduction, suggesting potential for clinical application against established infections. Although phages exhibit strong lytic activity against bacteria, the emergence of phage-resistant bacterial variants remains unavoidable. However, the development of phage resistance in bacteria often incurs fitness costs. In *K. pneumoniae*, for instance, mutations in capsular receptor-related genes that confer phage tolerance may simultaneously attenuate virulence-rendering the bacteria more susceptible to macrophage phagocytosis and/or increasing their antibiotic sensitivity (Fang et al. 2022).

In both our *G. mellonella* and mouse experiments, doses exceeding the optimal MOI were required to achieve effective therapeutic outcomes. This phenomenon has been consistently observed across multiple phage infection models, suggested that it represents a widespread and generalizable trend (Manohar et al. 2018). In the animal studies, phage Henu2_3 demonstrated significant efficacy in improving survival rates and reducing bacterial loads in organs. Notably, no phage-resistant bacterial mutants were observed in vivo, potentially attributable to the aforementioned virulence attenuation associated with phage resistance development. However, during clinical treatment, bacteria may develop resistance to phages targeting the same receptor by altering receptor synthesis, thus necessitating combination therapy using phages with distinct receptor specificities (Li et al. 2025). In *K. pneumoniae*, CPS acts as the dominant receptor for the majority of phages. Beyond CPS, other surface molecules-including LPS, the major porin OmpK36, outer membrane proteins OmpC and the siderophore receptor FepA-can also serve as functional receptors mediating phage adsorption and infection (Cai et al. 2022). Studies demonstrated that CPS mutations represent the principal driver of phage resistance evolution under both in vitro and in vivo conditions (Fu et al. 2025). Importantly, such mutations significantly reduced both fitness and virulence in hvKp strains. By designing phage cocktails targeting distinct receptors, we can not only broaden the therapeutic spectrum of phages but also effectively prevent the emergence of resistant bacterial strains (Yoo et al. 2024). Both oral and intravenous administration of engineered phage cocktails demonstrated significant efficacy in reducing *K. pneumoniae* colonization burden while concurrently ameliorating hepatic inflammation and disease pathology in susceptible SPF mice with hepatobiliary injury predisposition (Ichikawa et al. 2023). Furthermore, the feasibility of orally administered combination phage therapy in avoiding resistance, while effectively inhibited non-communicable disease-contributing pathobionts (Federici et al. 2022). Extensive systematic research and clinical studies on bacteriophages have laid the foundation for their application in combating MDR bacterial infections. Phage therapy is increasingly becoming a strategic imperative as an alternative to antibiotics for treating resistant bacterial infections.

## Conclusions

In this study, the isolated lytic bacteriophage, Henu2_3, effective against hvKp. This phage demonstrated remarkable stability across wide temperature (4–60℃) and pH (4–12) ranges, and maintained lytic activity in serum-containing environments. In vitro characterization revealed that Henu2_3 rapidly adsorbs to host bacteria, effectively inhibits hvKP growth, and disrupts mature biofilms. In vivo experiments showed the phage Henu2_3 significantly reduced bacterial loads in infected animals and improved survival rates. Whole genome sequencing confirmed the absence of antibiotic resistance or virulence genes in the genome of phage Henu2_3. These collective findings position Henu2_3 as an ideal candidate for inclusion in phage cocktails targeting *K. pneumoniae* infections.

## Electronic supplementary material

Below is the link to the electronic supplementary material.

Supplementary Material 1

## Data Availability

All data analyzed in this study are included in the published article and its supplementary information files, and further inquiries can be directed to the corresponding author. The complete genome sequence of phage Henu2_3 has been deposited in GenBank under the accession number PQ394119.1.

## Abbreviations

ORFs: Open reading frames
CFU: Colony-forming units
CPS: Capsular polysaccharides
MOI: Multiplicity of infection
OD: Optical density
TEM: Transmission electron microscopy
NCBI: National Center for Biotechnology Information
BLAST: Basic local alignment search tool
hvKp: Hypervirulent *Klebsiella pneumoniae*
PFU: Plaque-forming units
