# Supplementary material for: Characterization and genomics of phage Henu2_3 against K1 Klebsiella pneumoniae and its efficacy in animal models
Source: AMB Express. 2025 Jul 30;15:112. doi: 10.1186/s13568-025-01919-0 (PMC12311074; doi:10.1186/s13568-025-01919-0)

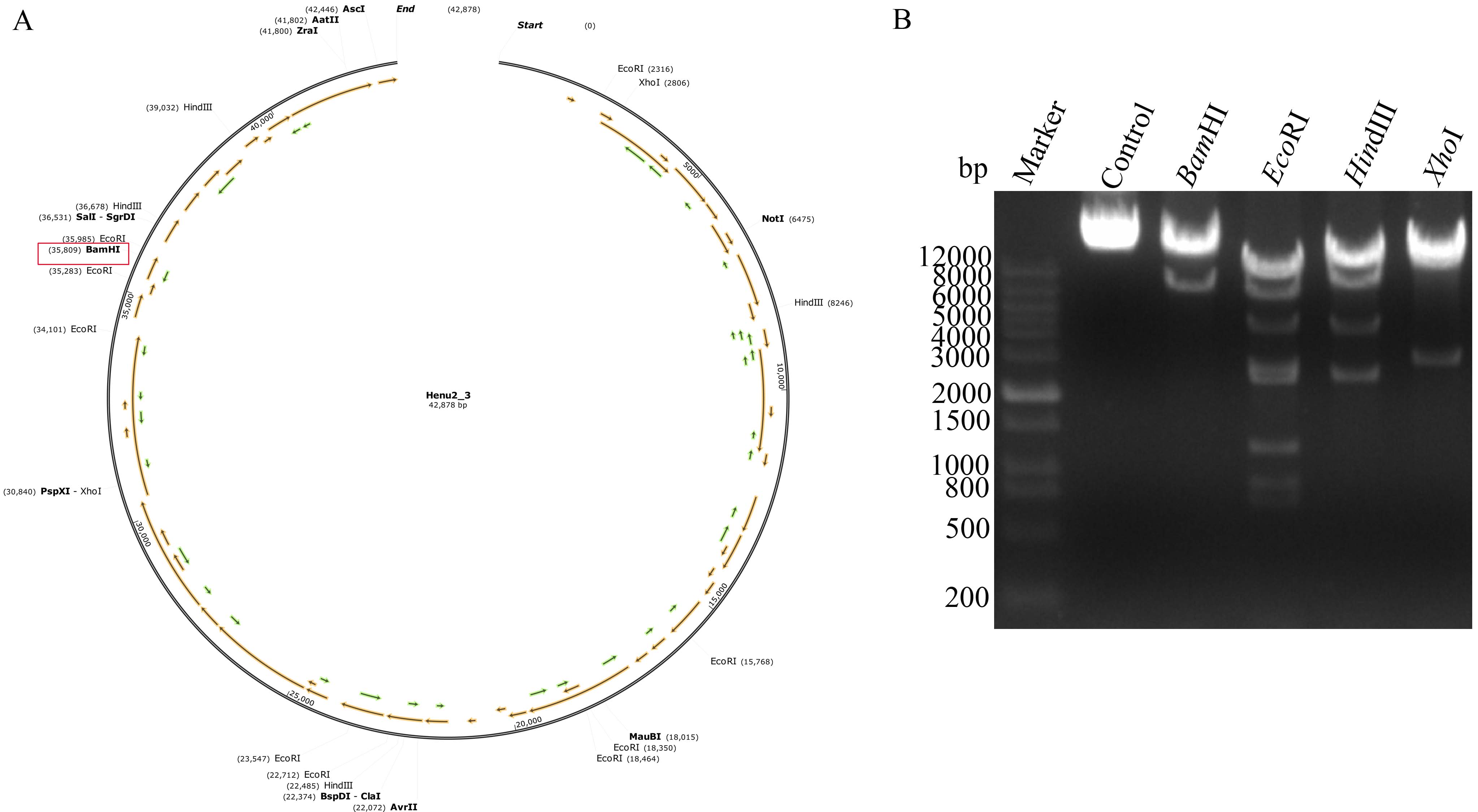


**Figure S1.** (A) The genomic DNA of phage Henu2_3 and its restriction enzyme sites were visualized using SnapeGene software. (B) Genomic DNA of phage Henu2_3 was subjected to restriction digestion by four restriction enzymes, namely, *Bam*HⅠ, *Eco*RⅠ, *Hin*dⅢ and *Xho*Ⅰ.


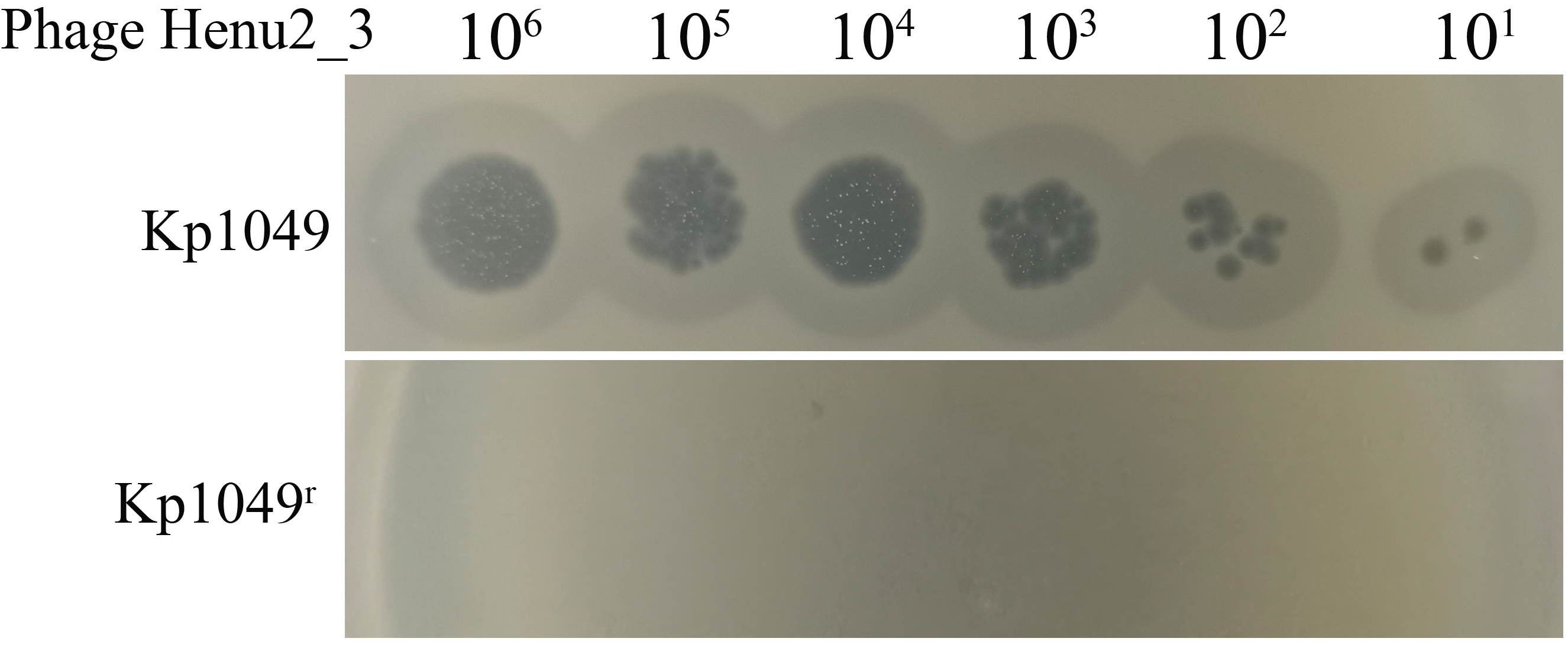


**Figure S2.** The spot test of phage Henu2_3 on the lawns of *K. pneumoniae* Kp1049 and Kp1049^r^.

>*Klebsiella pneumoniae* Kp1049 16S rRNA

GTGCCTGTCTCGCGGAAGCACAGAGAGCTTGCTCTCGGGTGACGAGCGGCGGACGGGTGAGTAATGTCTGGGAAACTGCCTGATGGAGGGGGATAACTACTGGAAACGGTAGCTAATACCGCATAATGTCGCAAGACCAAAGTGGGGGACCTTCGGGCCTCATGCCATCAGATGTGCCCAGATGGGATTAGCTAGTAGGTGGGGTAACGGCTCACCTAGGCGACGATCCCTAGCTGGTCTGAGAGGATGACCAGCCACACTGGAACTGAGACACGGTCCAGACTCCTACGGGAGGCAGCAGTGGGGAATATTGCACAATGGGCGCAAGCCTGATGCAGCCATGCCGCGTGTGTGAAGAAGGCCTTCGGGTTGTAAAGCACTTTCAGCGGGGAGGAAGGCGATAAGGTTAATAACCTTGGCGATTGACGTTACCCGCAGAAGAAGCACCGGCTAACTCCGTGCCAGCAGCCGCGGTAATACGGAGGGTGCAAGCGTTAATCGGAATTACTGGGCGTAAAGCGCACGCAGGCGGTCTGTCAAGTCGAATGTGAAATCCCCCGGGCTCAACCTGGGAACTGCATTCGAAACTGGCAGGCTAGAGTCTTGTAGAGGGGGGTAGAATTTCCAGGTGTAGCGGTGAAATGCGTAGAGATCTGGAGGAATACCGGTGGCGAAGGCGGCCCCCTGGACAAAGACTGACGCTCAGGTGCGAAAGCGTGGGGAGCAAACAGGATTAGATACCCCTGGTAGTCCACGCCGTAAACGATGTCGAATTTTGGAGGTTGTGCCCCTTGAGGCGTGGGCTTCCGGAAGCTAACGCGTTAAATCGACCGGCCTGGGGGAGTACCGGCCCGCAAGGGTTAAAACTCCAAAGGAAATTGACGGGGGCCCGCACAAGCGGTGGAGCATGTGGTTTAATTCGATGCAACGCGAAGAACCTTACCTGGTCTTGACATCCACAGAACTTTCCAGAGATGGATTGGTGCCTTCGGGAACTGTGAGACAGGTGCTGCATGGCTGTCGTCAGCTCGTGTTGTGAAATGTTGGGTTAAGTCCCGCAACGAGCGCAACCCTTATCCTTTGTTGCCAGCGGTTCGGCCGGGAACTCAAAGGAGACTGCCAGTGATAAACTGGAGGAAGGTGGGGATGACGTCAAGTCATCATGGCCCTTACGACCAGGGCTACACACGTGCTACAATGGCATATACAAAGAGAAGCGACCTCGCGAGAGCAAGCGGACCTCATAAAGTATGTCGTAGTCCGGATTGGAGTCTGCAACTCGACTCCATGAAGTCGGAATCGCTAGTAATCGTAGATCAGAATGCTACGGTGAATACGTTCCCGGGCCTTGTACACACCGCCCGTCACACCATGGGAGTGGGTTGCAAAAGAAGTAGGCAGCTCAAAGGT

The ethical approval certificate for conducting this study


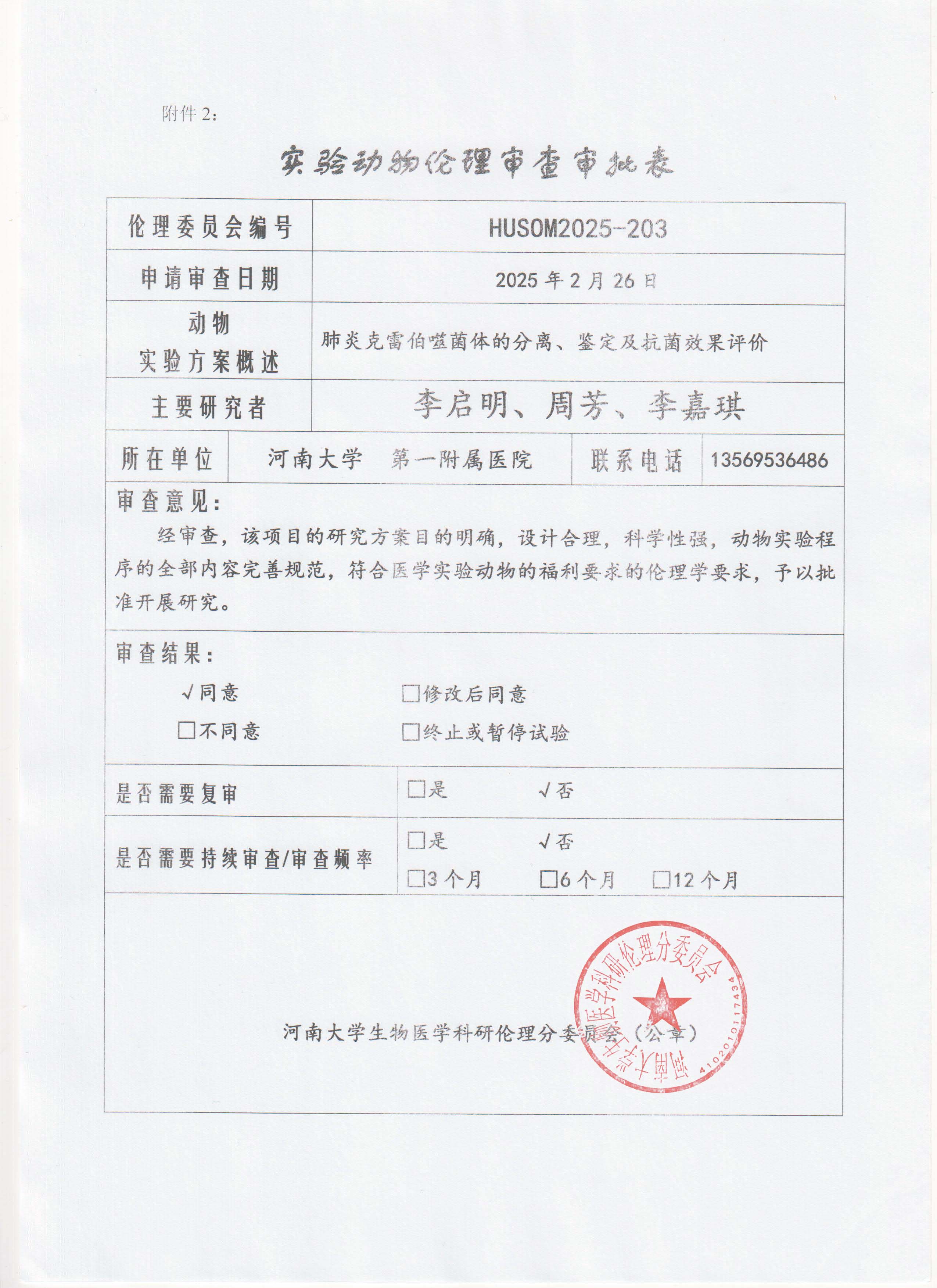

Supplement: Supplementary file 1 — Supplementary Material 1 [file 13568_2025_1919_MOESM1_ESM.doc]
